# Exaggerated Arthropod Bite: A Case Report and Review of the Mimics

**DOI:** 10.5811/cpcem.2017.12.37034

**Published:** 2018-01-11

**Authors:** Sagah Ahmed, Elaine Bromberek, Joelle Borhart

**Affiliations:** * George Washington University School of Medicine and Health Sciences, Washington, District of Columbia; † MedStar Georgetown University Hospital, Department of Emergency Medicine, Washington, District of Columbia

## Abstract

Exaggerated arthropod bite reactions causing hemorrhagic or necrotic bullous lesions can mimic other serious conditions such as cutaneous anthrax, brown recluse spider bite, and tularemia. A 55- year-old, healthy woman presented to the emergency department with a 3.5-centimeter painless, collapsed hemorrhagic bulla at the left costal margin. She was afebrile and had no systemic symptoms. Laboratory evaluation was unremarkable. She was prescribed silver sulfadiazine cream and mupirocin ointment. The area denuded two days later and the lesion completely healed. This case illustrates the broad differential to be considered when evaluating patients with hemorrhagic bullous lesions.

## INTRODUCTION

Dermatologic complaints make up to 5–8% of emergency department (ED) visits in the United States every year.^1^ Patients frequently present to the ED for evaluation of a “bug bite,” particularly if their reaction is atypical or dramatic. Although arthropod bites usually result in mild and self-limited symptoms, exaggerated arthropod bite reactions causing hemorrhagic or necrotic bullous lesions can mimic other serious conditions such as cutaneous anthrax, brown recluse spider bite, or tularemia. The following case illustrates the wide differential that should be considered for a patient who presents to the ED with a necrotic bullous lesion.

## CASE REPORT

A 55-year-old, otherwise-healthy woman presented to the ED with complaint of a “possible bug bite.” Four days prior, she noticed a small, painless red lesion on her left abdomen. A day later, she noticed that the area had become increasingly red. Over the next 72 hours, the redness expanded and the center of the lesion became dark black in color. She also noticed redness surrounding the darkened area over the prior three days. She did not remember being bit by a spider or insect, but she is an avid gardener. She lives in Maine, had no significant recent travel, no sick contacts, no contact with non-domesticated animals, and denied systemic symptoms. She reported mild pruritus at the site but no pain.

**Image 1:**
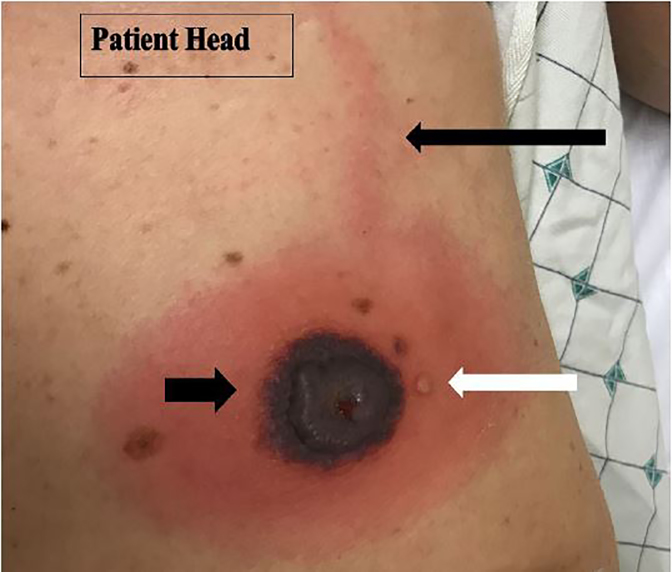
Patient lesion. Approximately three-to-four centimeter, collapsed hemorrhagic bulla with two-centimeter, surrounding indurated, non-tender erythema (short black arrow). Immediately adjacent to the large bulla, a two- millimeter, clear vesicle is present (white arrow). A linear erythematous patch extended cranially from the superior aspect of the large bulla and surrounding erythema (long black arrow).

On physical exam, the patient was afebrile and well appearing with normal vital signs. On her left abdomen at the costal margin there was a 3.5-centimeter (cm), collapsed hemorrhagic bulla and surrounding indurated, non-tender erythema up to two cm from the central bulla. Immediately adjacent to the large bulla, a two-millimeter (mm), clear vesicle was present. A linear erythematous streak extended cranially from the superior aspect of the large bulla (Image 1).

Laboratory studies revealed a white blood cell count of 8,900/mm^3^ with 81% neutrophils and 10% lymphocytes and an otherwise-normal differential. Electrolytes, liver function tests and coagulation panel were normal. Gram stain showed moderate white blood cells, rare epithelial cells, and no organisms seen. A wound culture showed no growth at three days. Blood cultures were also obtained and were negative. Dermatology was consulted and determined that the patient most likely had an exaggerated arthropod bite reaction. She was prescribed silver sulfadiazine cream to the open area of the wound and mupirocin ointment. The area denuded two days later and the lesion completely healed.

## DISCUSSION

Typical arthropod bites cause minor, self-limited symptoms such as pruritic, pink papules only a few millimeters in size. Rarely, bites cause large immunologic reactions known as exaggerated arthropod bites. These can be large, erythematous, pruritic, edematous and sometimes hemorrhagic or necrotic. On histologic examination, they show a high number of eosinophils and a predominance of T- and B-cell lymphocytes.^2^

Most cases of exaggerated arthropod bites have been described in patients with chronic lymphocytic leukemia and other lymphoproliferative disorders, but patients with known allergies as well as healthy patients have developed similar reactions.^2^ In the absence of a lymphoproliferative disorder, the pathophysiology is related to sensitization of T cells throughout the patient’s life. Increased immunoglobulin E production in patients with exaggerated arthropod reactions may also be related. Care is primarily supportive, although topical antibiotics can be used to prevent superinfection.^3^

**Image 2:**
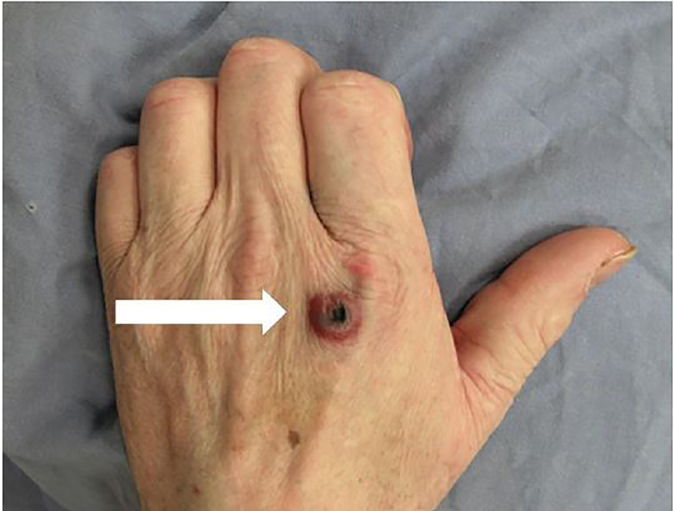
Necrotic wound with a hemorrhagic rim on the left hand of a patient with cutaneous anthrax (white arrow). From: Cinquetti G, Banal F, Dupuy A–L, et al. Three related cases of cutaneous anthrax in France. Medicine 2009;88:371. (With permission)

Exaggerated arthropod bites can mimic serious conditions such as cutaneous anthrax, brown recluse spider bite, and tularemia. Cutaneous anthrax lesions usually begin as a small, painless, and often pruritic papule that rapidly enlarges and develops a central vesicle or bulla in 24–36 hours; the vesicle then becomes necrotic and dried, leaving a characteristic painless black eschar surrounded by extensive edema in the neighboring tissues (Image 2).

CPC-EM Capsule

What do we already know about this clinical entity?

Exaggerated arthropod bite reactions tend to occur in patients with lymphoproliferative disorders and can mimic serious conditions such as cutaneous anthrax.

What makes this presentation of disease reportable?

This is a case of a healthy, immune-competent patient with an exaggerated arthropod bite reaction presenting with a hemorrhagic bullous lesion.

What is the major learning point?

Arthropod bite reactions can be atypical or dramatic, even in immune-competent patients.

How might this improve emergency medicine practice?

This case illustrates the wide differential that should be considered for a patient who presents to the emergency department for evaluation of a “bug bite.”

**Image 3:**
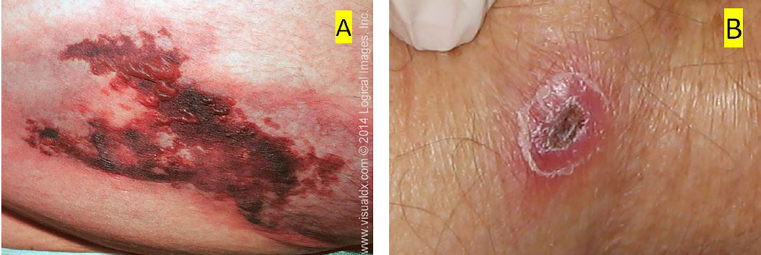
A) Brown recluse spider bite. From: www.visualdx.com, Logical Images, Inc. Graphic 96174, with permission, B) and tularemia, courtesy of Todd M Pollack, MD.

Brown recluse spiders are endemic in regions of the Central, South, and Midwestern United States. The initial bite is usually painless and begins as a red plaque that often develops central pallor with occasional surrounding vesiculation (Image 3A).

The lesion becomes increasingly painful over the next two to eight hours. In most cases, the wound is self-limited and resolves without further complications. In less than 10% of cases, however, necrosis will occur within 48–72 hours and the lesion subsequently breaks down into an ulcerating eschar.^4^ Tularemia is a zoonotic infection caused by the aerobic gram-negative coccobacillus, *Francisella tularensis*. Patients usually present with fever and a single erythematous ulcerative lesion with a central eschar at the site of a bite; however, more than one skin lesion may be present (Image 3B).

## CONCLUSION

Patients frequently present to the ED with “bug bites.” Exaggerated arthropod bite reactions are worrisome as they can mimic serious conditions. Emergency physicians must be prepared to use a combination of history, physical exam, geographic location, epidemiologic history, and laboratory results to make a timely and accurate diagnosis.
